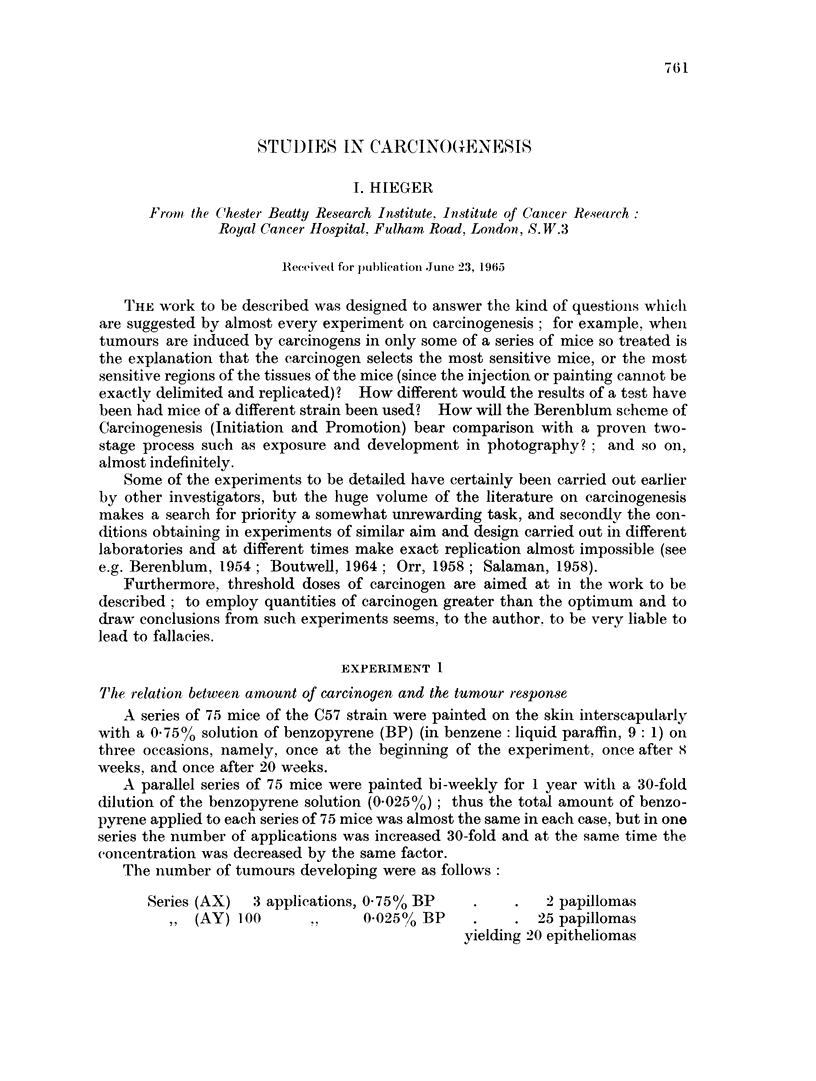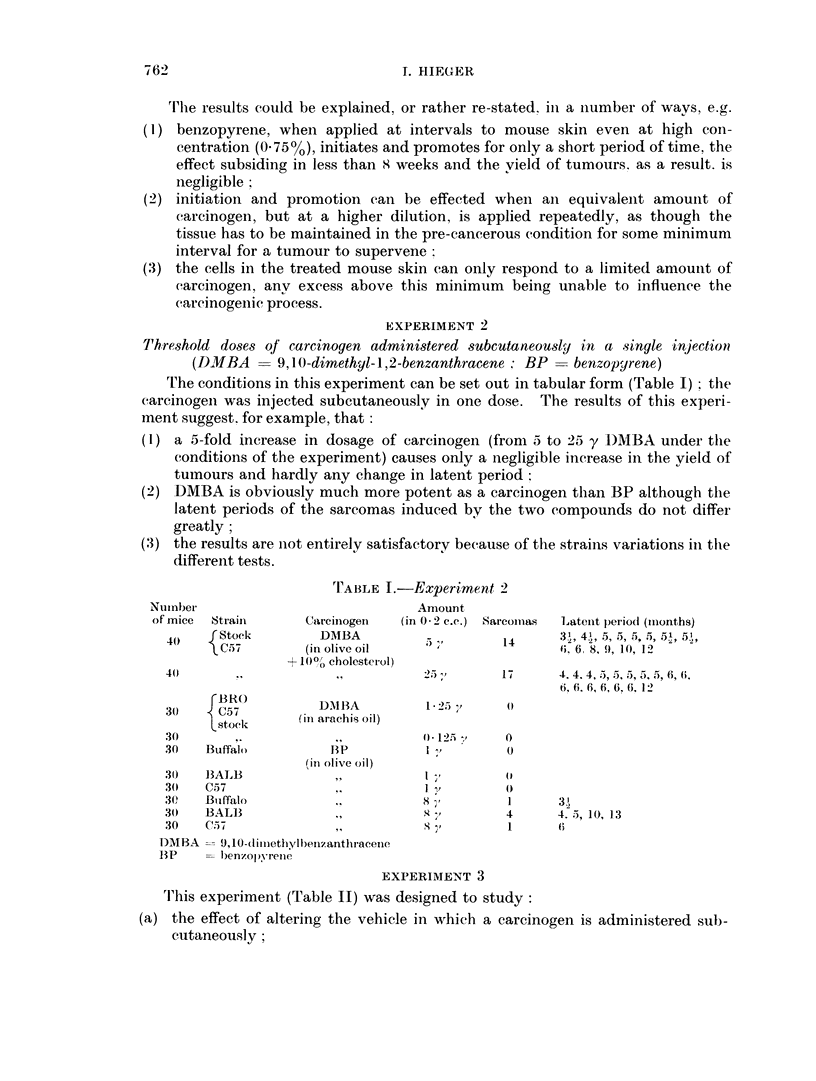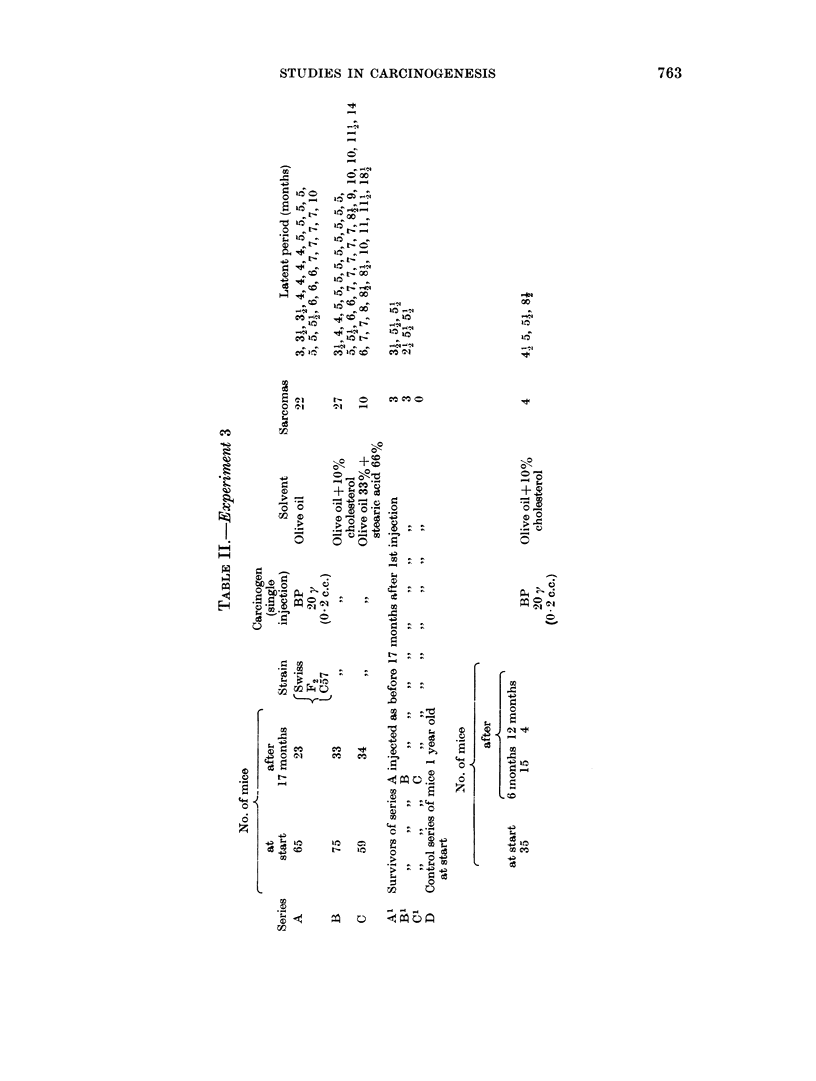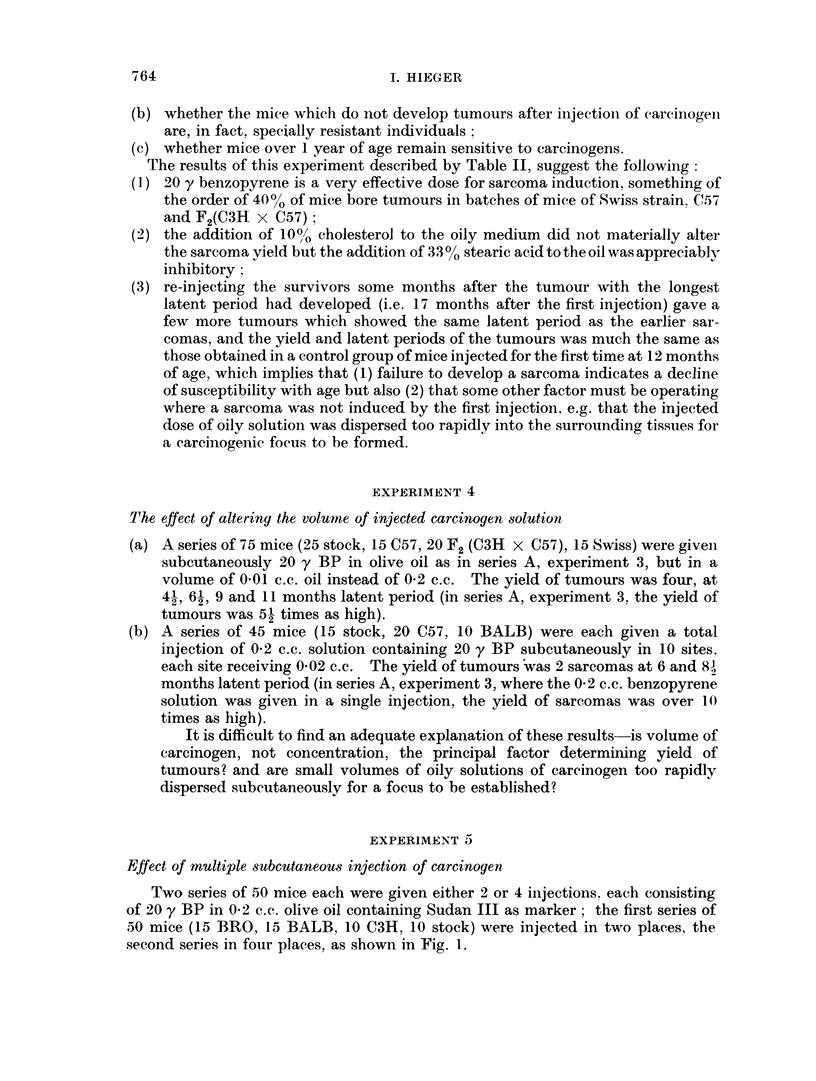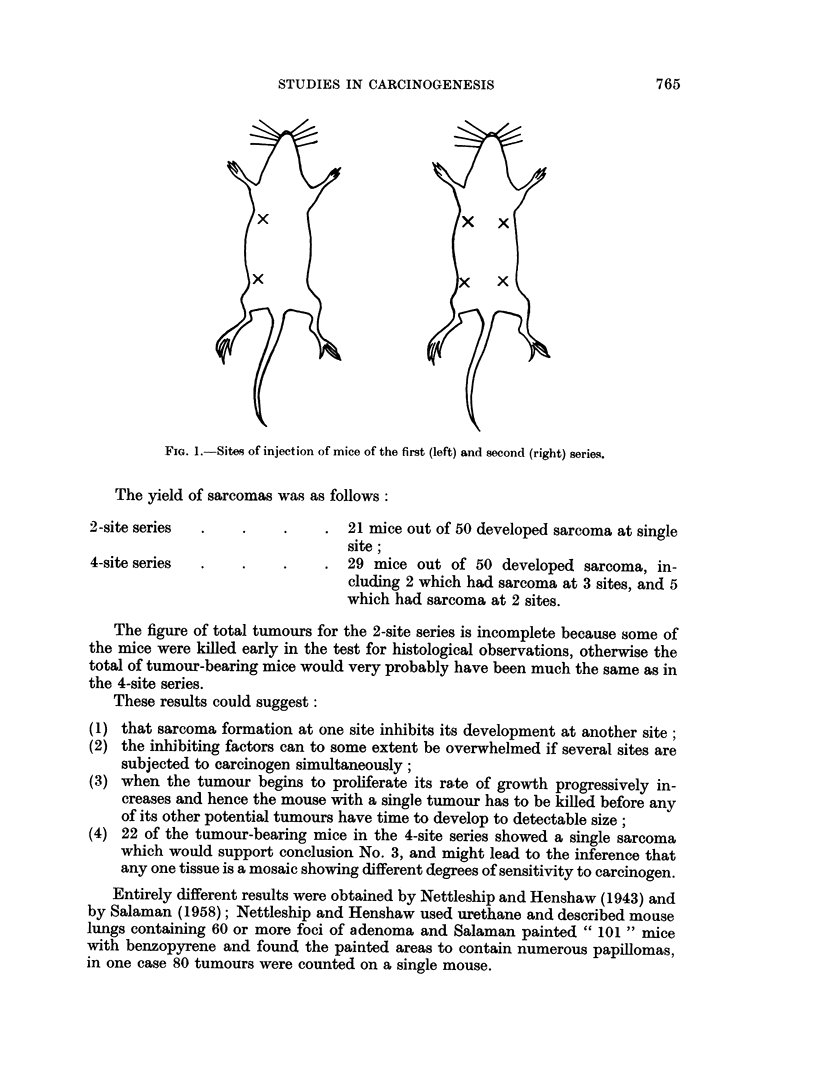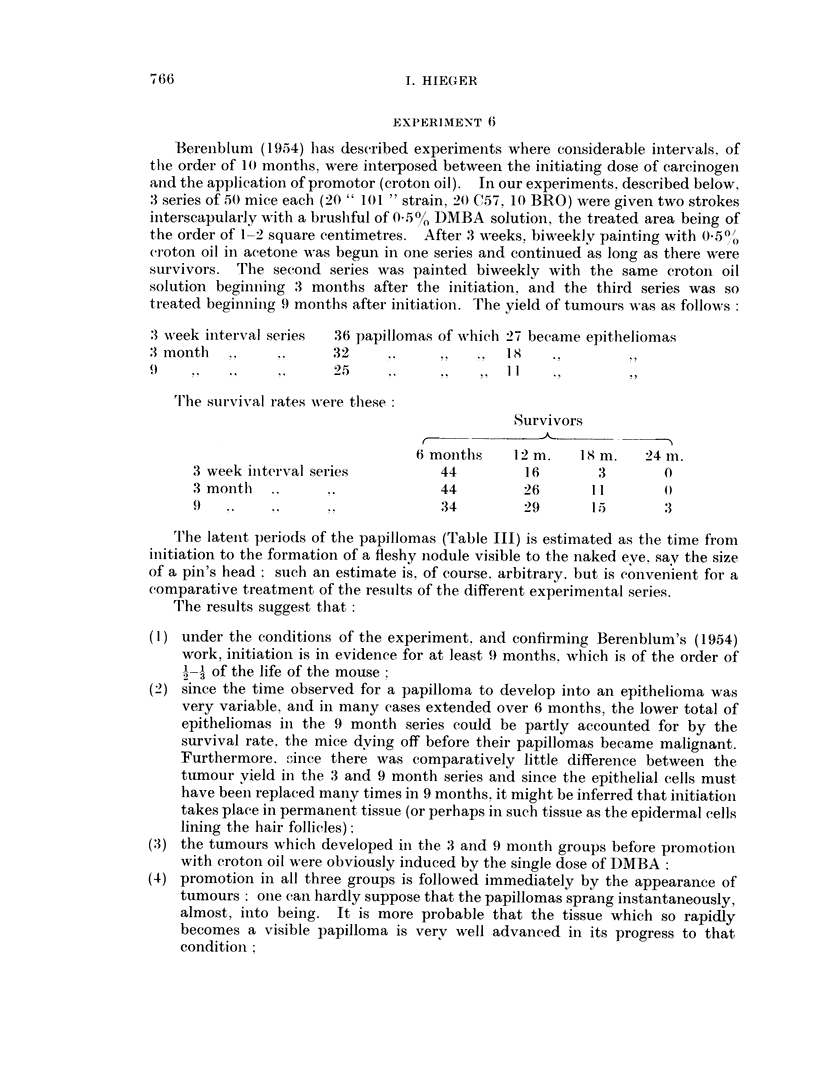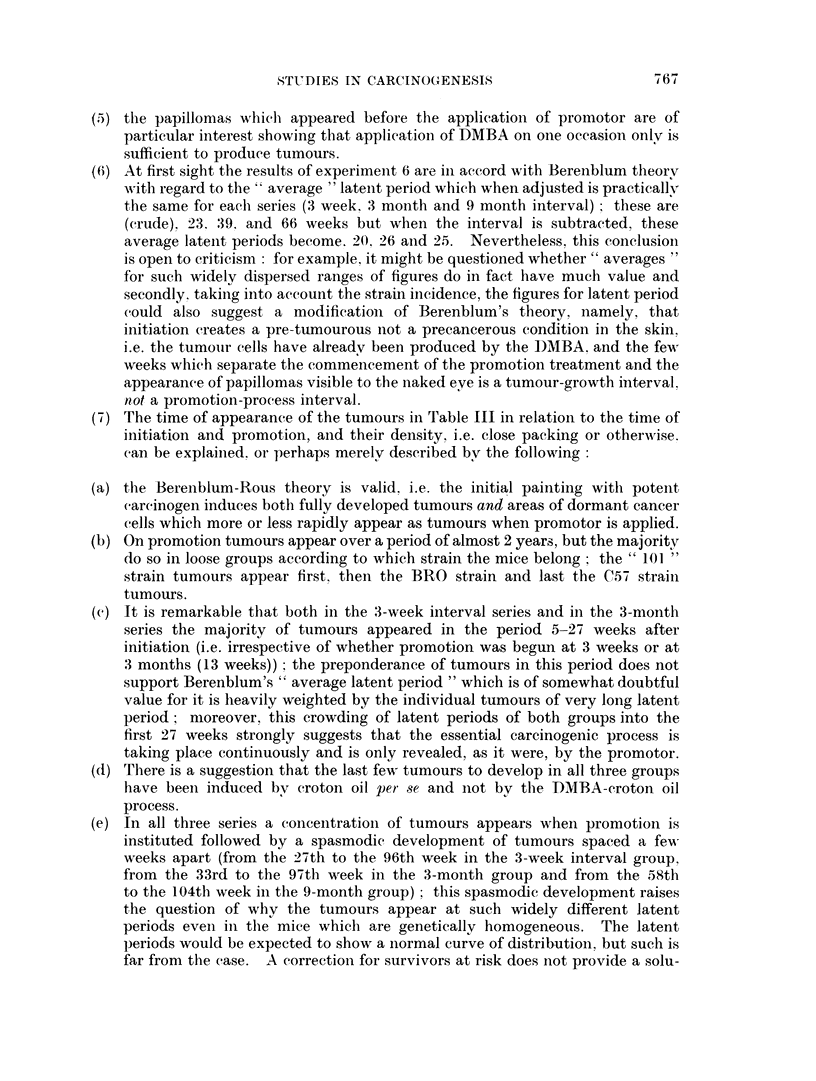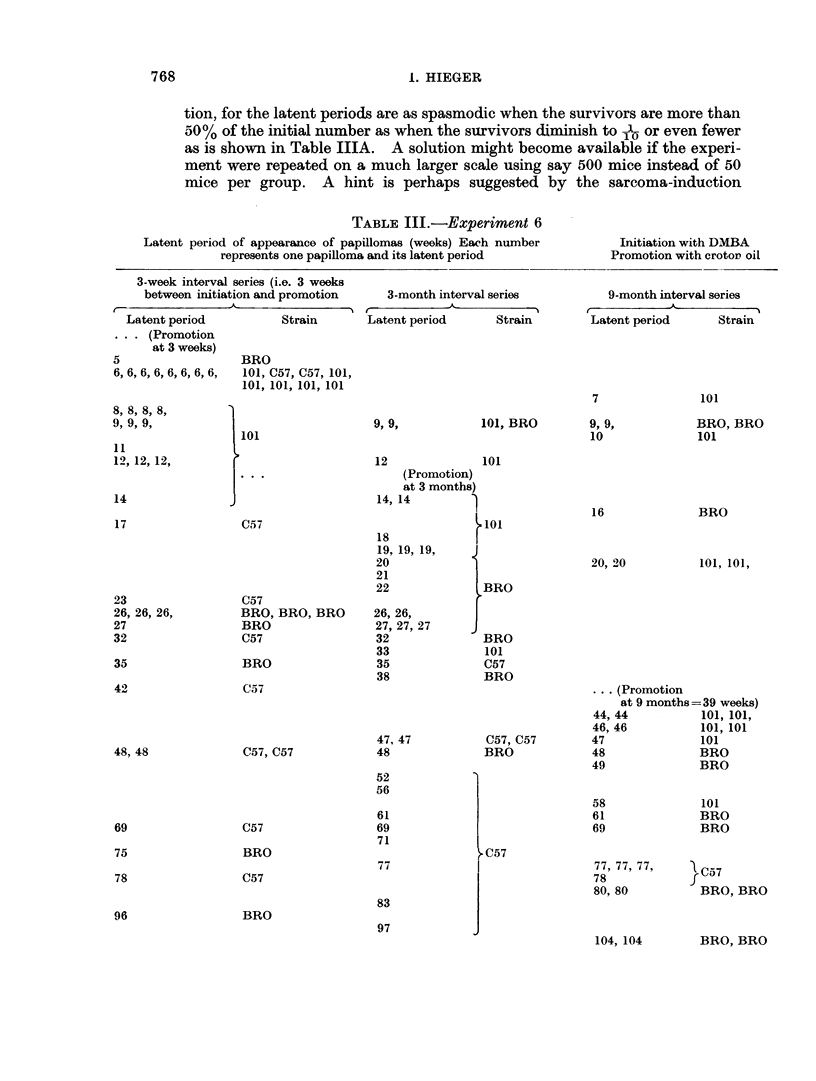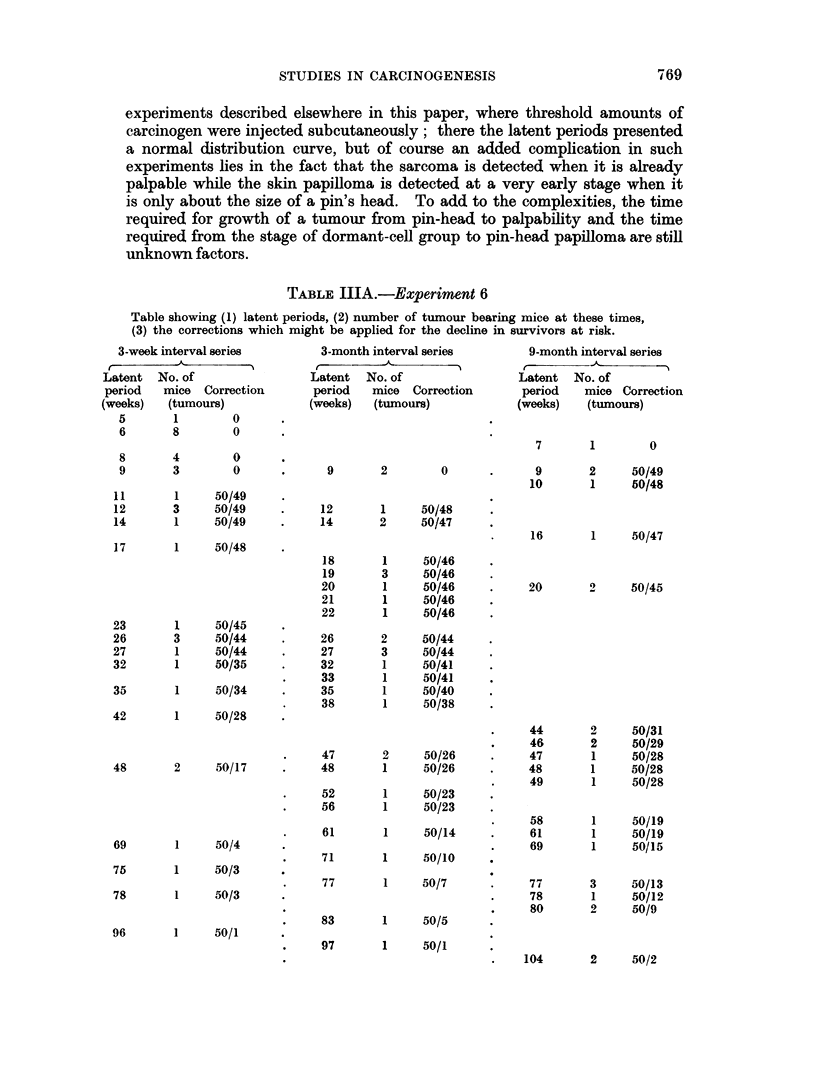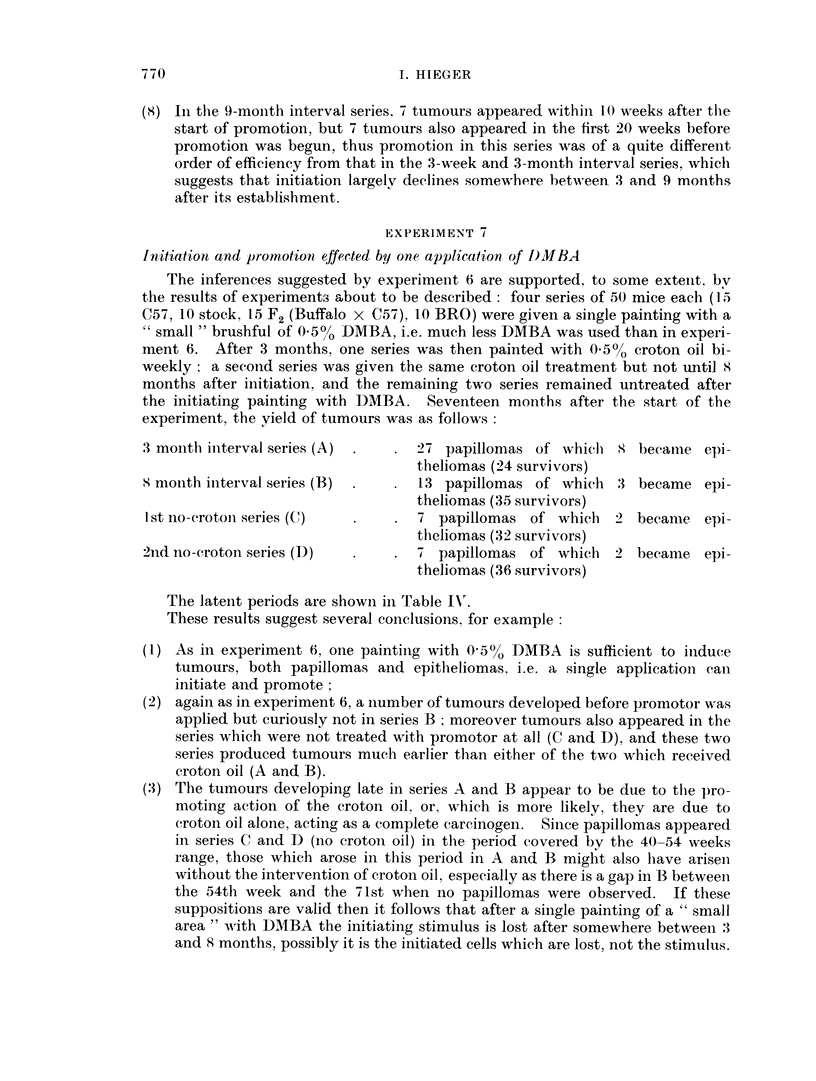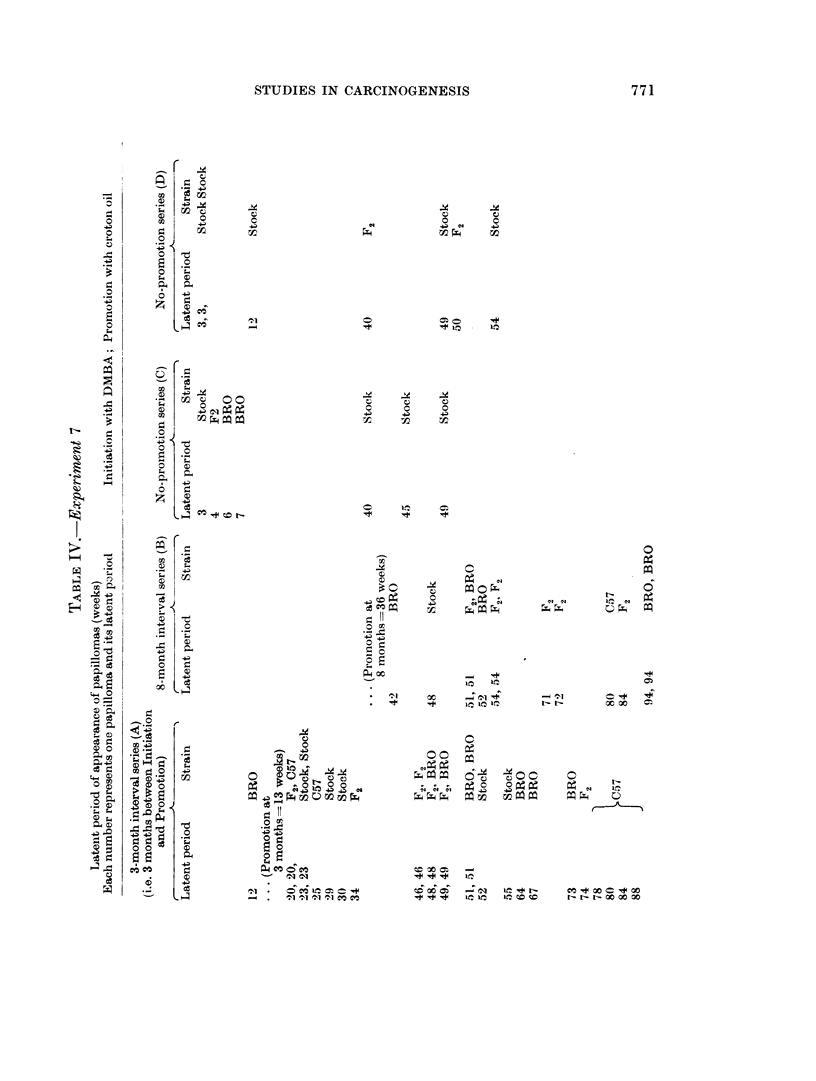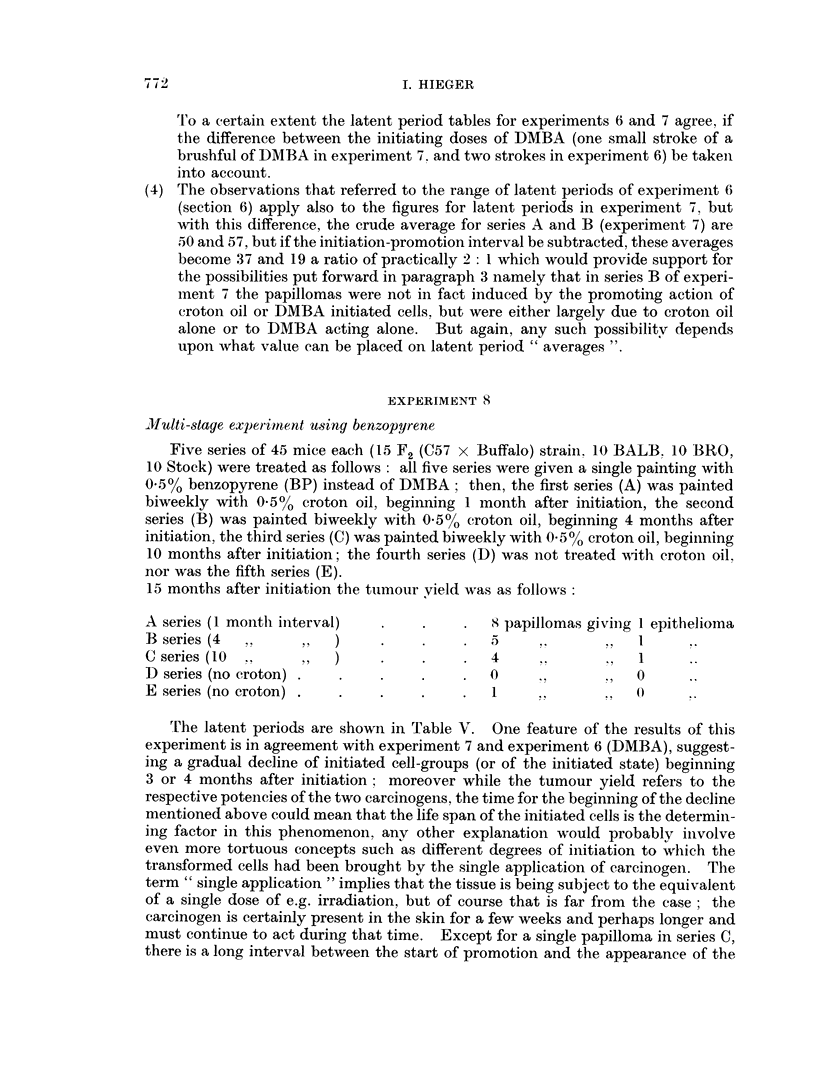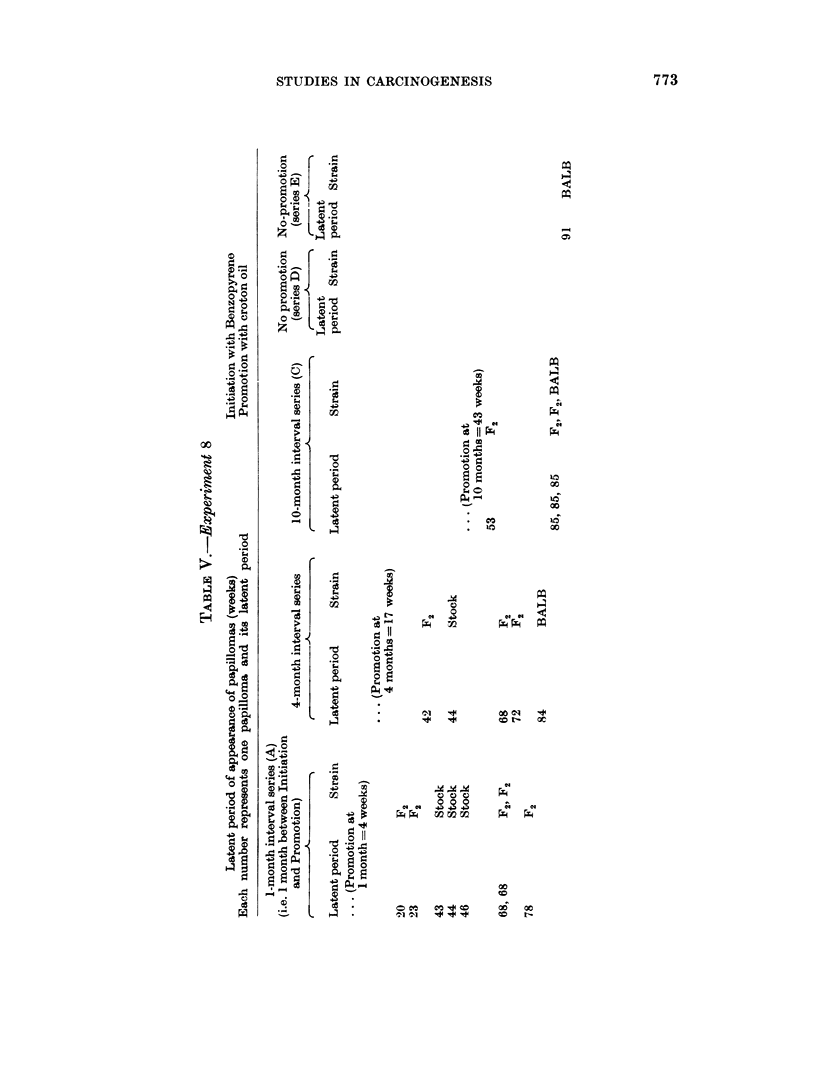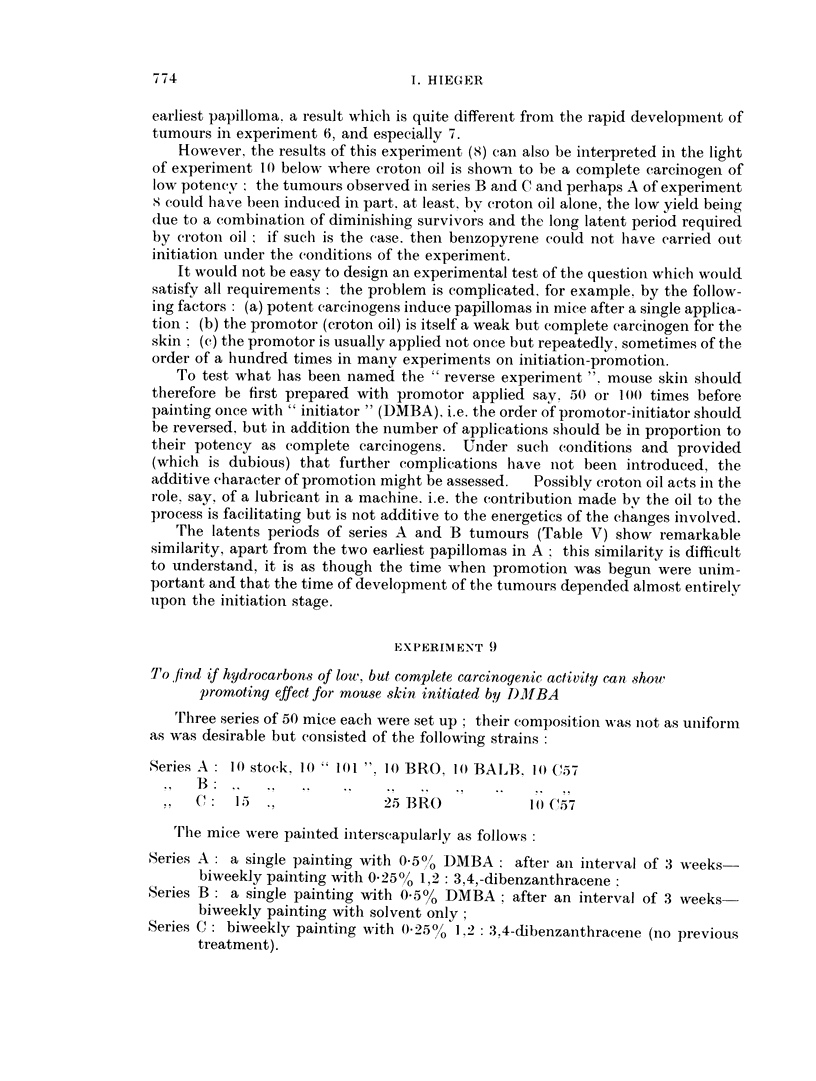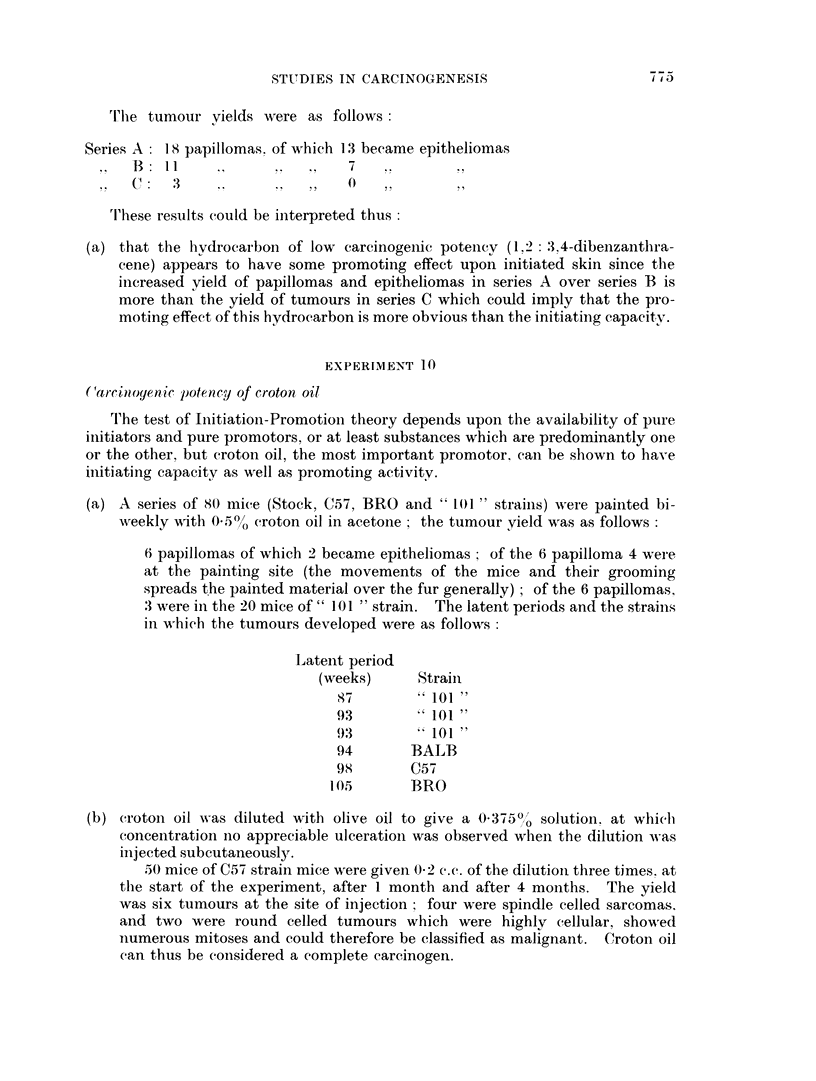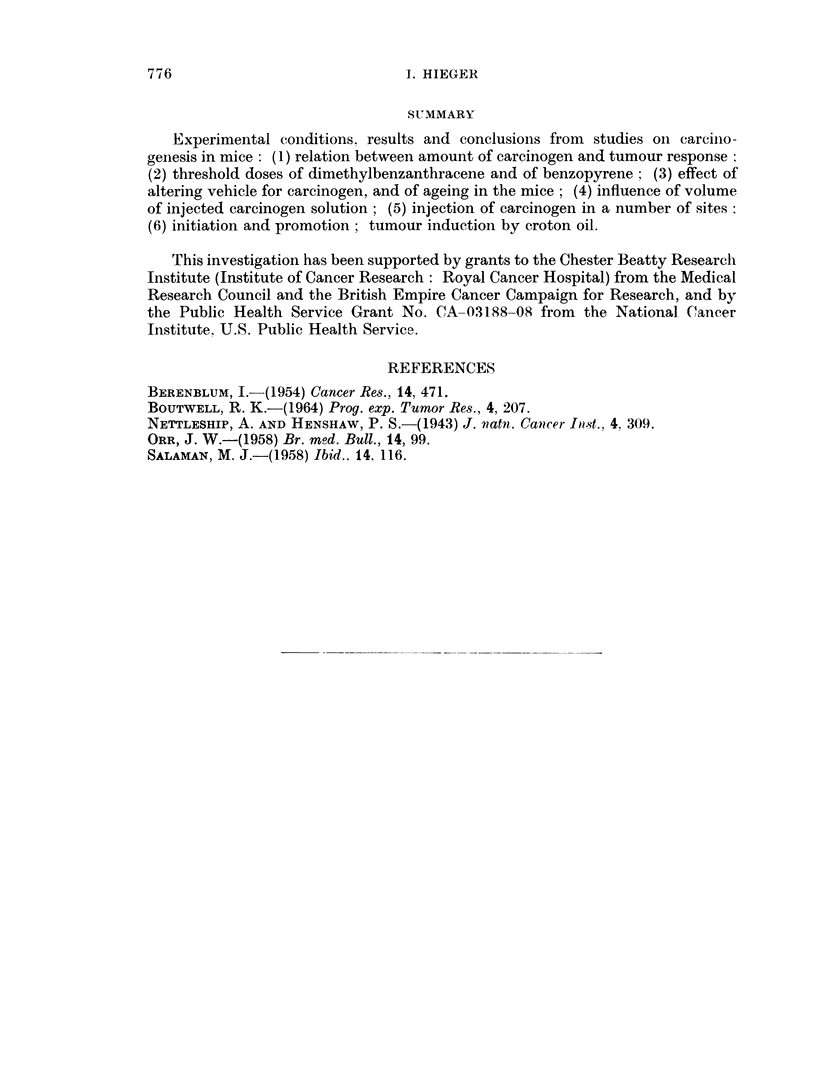# Studies in carcinogenesis.

**DOI:** 10.1038/bjc.1965.89

**Published:** 1965-12

**Authors:** I. Hieger


### STUDIES IN CARCINOGENESIS

I. HIEGER

*From the Chester Beatty Research Institute, Institute of Cancer Research : Royal Cancer Hospital, Fulham Road, London, S.W.3*

Received for publication June 23, 1965

THE work to be described was designed to answer the kind of questions which are suggested by almost every experiment on carcinogenesis ; for example, when tumours are induced by carcinogens in only some of a series of mice so treated is the explanation that the carcinogen selects the most sensitive mice, or the most sensitive regions of the tissues of the mice (since the injection or painting cannot be exactly delimited and replicated)? How different would the results of a test have been had mice of a different strain been used? How will the Berenblum scheme of Carcinogenesis (Initiation and Promotion) bear comparison with a proven two-stage process such as exposure and development in photography? ; and so on, almost indefinitely.

Some of the experiments to be detailed have certainly been carried out earlier by other investigators, but the huge volume of the literature on carcinogenesis makes a search for priority a somewhat unrewarding task, and secondly the conditions obtaining in experiments of similar aim and design carried out in different laboratories and at different times make exact replication almost impossible (see e.g. Berenblum, 1954 ; Boutwell, 1964 ; Orr, 1958 ; Salaman, 1958).

Furthermore, threshold doses of carcinogen are aimed at in the work to be described ; to employ quantities of carcinogen greater than the optimum and to draw conclusions from such experiments seems, to the author, to be very liable to lead to fallacies.

### EXPERIMENT 1

*The relation between amount of carcinogen and the tumour response*

A series of 75 mice of the C57 strain were painted on the skin interscapularly with a 0·75% solution of benzopyrene (BP) (in benzene : liquid paraffin, 9 : 1) on three occasions, namely, once at the beginning of the experiment, once after 8 weeks, and once after 20 weeks.

A parallel series of 75 mice were painted bi-weekly for 1 year with a 30-fold dilution of the benzopyrene solution (0·025%) ; thus the total amount of benzopyrene applied to each series of 75 mice was almost the same in each case, but in one series the number of applications was increased 30-fold and at the same time the concentration was decreased by the same factor.

The number of tumours developing were as follows :

| | | | |
|---|---|---|---|
| Series (AX) | 3 applications, | 0·75% BP | . . 2 papillomas |
| ,, (AY) | 100 ,, | 0·025% BP | . . 25 papillomas yielding 20 epitheliomas |

The results could be explained, or rather re-stated, in a number of ways, e.g.

(1) benzopyrene, when applied at intervals to mouse skin even at high concentration (0·75%), initiates and promotes for only a short period of time, the effect subsiding in less than 8 weeks and the yield of tumours, as a result, is negligible ;

(2) initiation and promotion can be effected when an equivalent amount of carcinogen, but at a higher dilution, is applied repeatedly, as though the tissue has to be maintained in the pre-cancerous condition for some minimum interval for a tumour to supervene ;

(3) the cells in the treated mouse skin can only respond to a limited amount of carcinogen, any excess above this minimum being unable to influence the carcinogenic process.

### EXPERIMENT 2

*Threshold doses of carcinogen administered subcutaneously in a single injection (DMBA = 9,10-dimethyl-1,2-benzanthracene ; BP = benzopyrene)*

The conditions in this experiment can be set out in tabular form (Table I) ; the carcinogen was injected subcutaneously in one dose. The results of this experiment suggest, for example, that :

(1) a 5-fold increase in dosage of carcinogen (from 5 to 25 $\gamma$ DMBA under the conditions of the experiment) causes only a negligible increase in the yield of tumours and hardly any change in latent period ;

(2) DMBA is obviously much more potent as a carcinogen than BP although the latent periods of the sarcomas induced by the two compounds do not differ greatly ;

(3) the results are not entirely satisfactory because of the strains variations in the different tests.

TABLE I.—*Experiment 2*

| Number of mice | Strain | Carcinogen | Amount (in 0·2 c.c.) | Sarcomas | Latent period (months) |
|---|---|---|---|---|---|
| 40 | Stock, C57 | DMBA (in olive oil + 10% cholesterol) | 5 $\gamma$ | 14 | $3\frac{1}{2}$, $4\frac{1}{2}$, 5, 5, 5, 5, $5\frac{1}{2}$, $5\frac{1}{2}$, 6, 6, 8, 9, 10, 12 |
| 40 | ,, | ,, | 25 $\gamma$ | 17 | 4, 4, 4, 5, 5, 5, 5, 5, 6, 6, 6, 6, 6, 6, 6, 6, 12 |
| 30 | BRO, C57, stock | DMBA (in arachis oil) | 1·25 $\gamma$ | 0 | |
| 30 | ,, | ,, | 0·125 $\gamma$ | 0 | |
| 30 | Buffalo | BP (in olive oil) | 1 $\gamma$ | 0 | |
| 30 | BALB | ,, | 1 $\gamma$ | 0 | |
| 30 | C57 | ,, | 1 $\gamma$ | 0 | |
| 30 | Buffalo | ,, | 8 $\gamma$ | 1 | $3\frac{1}{2}$ |
| 30 | BALB | ,, | 8 $\gamma$ | 4 | 4, 5, 10, 13 |
| 30 | C57 | ,, | 8 $\gamma$ | 1 | 6 |

DMBA = 9,10-dimethylbenzanthracene
BP = benzopyrene

### EXPERIMENT 3

This experiment (Table II) was designed to study :

(a) the effect of altering the vehicle in which a carcinogen is administered subcutaneously ;

TABLE II.—*Experiment* 3

| Series | No. of mice: at start | No. of mice: after 17 months | Strain | Carcinogen (single injection) | Solvent | Sarcomas | Latent period (months) |
|---|---|---|---|---|---|---|---|
| A | 65 | 23 | Swiss, $F_2$, C57 | BP 20 $\gamma$ (0·2 c.c.) | Olive oil | 22 | 3, $3\frac{1}{2}$, $3\frac{1}{2}$, 4, 4, 4, 4, 5, 5, 5, 5, 5, 5, $5\frac{1}{2}$, 6, 6, 6, 7, 7, 7, 7, 10 |
| B | 75 | 33 | ,, | ,, | Olive oil+10% cholesterol | 27 | $3\frac{1}{2}$, 4, 4, 5, 5, 5, 5, 5, 5, 5, 5, 5, 5, $5\frac{1}{2}$, 6, 6, 7, 7, 7, 7, 7, $8\frac{1}{2}$, 9, 10, 10, $11\frac{1}{2}$, 14 |
| C | 59 | 34 | ,, | ,, | Olive oil 33%+ stearic acid 66% | 10 | 6, 7, 7, 8, $8\frac{1}{2}$, $8\frac{1}{2}$, 10, 11, $11\frac{1}{2}$, $18\frac{1}{2}$ |
| A¹ | Survivors of series A injected as before 17 months after 1st injection | | | | | 3 | $3\frac{1}{2}$, $5\frac{1}{2}$, $5\frac{1}{2}$ |
| B¹ | ,, ,, ,, B ,, ,, ,, ,, ,, ,, ,, ,, | | | | | 3 | $2\frac{1}{2}$ $5\frac{1}{2}$ $5\frac{1}{2}$ |
| C¹ | ,, ,, ,, C ,, ,, ,, ,, ,, ,, ,, ,, | | | | | 0 | |
| D | Control series of mice 1 year old at start. No. of mice: at start 35 | after 6 months 15; after 12 months 4 | | BP 20 $\gamma$ (0·2 c.c.) | Olive oil+10% cholesterol | 4 | $4\frac{1}{2}$ 5, $5\frac{1}{2}$, $8\frac{1}{2}$ |

(b) whether the mice which do not develop tumours after injection of carcinogen are, in fact, specially resistant individuals ;

(c) whether mice over 1 year of age remain sensitive to carcinogens.

The results of this experiment described by Table II, suggest the following :

(1) 20 $\gamma$ benzopyrene is a very effective dose for sarcoma induction, something of the order of 40% of mice bore tumours in batches of mice of Swiss strain, C57 and $F_2$(C3H $\times$ C57) ;

(2) the addition of 10% cholesterol to the oily medium did not materially alter the sarcoma yield but the addition of 33% stearic acid to the oil was appreciably inhibitory ;

(3) re-injecting the survivors some months after the tumour with the longest latent period had developed (i.e. 17 months after the first injection) gave a few more tumours which showed the same latent period as the earlier sarcomas, and the yield and latent periods of the tumours was much the same as those obtained in a control group of mice injected for the first time at 12 months of age, which implies that (1) failure to develop a sarcoma indicates a decline of susceptibility with age but also (2) that some other factor must be operating where a sarcoma was not induced by the first injection, e.g. that the injected dose of oily solution was dispersed too rapidly into the surrounding tissues for a carcinogenic focus to be formed.

### EXPERIMENT 4

*The effect of altering the volume of injected carcinogen solution*

(a) A series of 75 mice (25 stock, 15 C57, 20 $F_2$ (C3H $\times$ C57), 15 Swiss) were given subcutaneously 20 $\gamma$ BP in olive oil as in series A, experiment 3, but in a volume of 0·01 c.c. oil instead of 0·2 c.c. The yield of tumours was four, at $4\frac{1}{2}$, $6\frac{1}{2}$, 9 and 11 months latent period (in series A, experiment 3, the yield of tumours was $5\frac{1}{2}$ times as high).

(b) A series of 45 mice (15 stock, 20 C57, 10 BALB) were each given a total injection of 0·2 c.c. solution containing 20 $\gamma$ BP subcutaneously in 10 sites, each site receiving 0·02 c.c. The yield of tumours was 2 sarcomas at 6 and $8\frac{1}{2}$ months latent period (in series A, experiment 3, where the 0·2 c.c. benzopyrene solution was given in a single injection, the yield of sarcomas was over 10 times as high).

It is difficult to find an adequate explanation of these results—is volume of carcinogen, not concentration, the principal factor determining yield of tumours? and are small volumes of oily solutions of carcinogen too rapidly dispersed subcutaneously for a focus to be established?

### EXPERIMENT 5

*Effect of multiple subcutaneous injection of carcinogen*

Two series of 50 mice each were given either 2 or 4 injections, each consisting of 20 $\gamma$ BP in 0·2 c.c. olive oil containing Sudan III as marker ; the first series of 50 mice (15 BRO, 15 BALB, 10 C3H, 10 stock) were injected in two places, the second series in four places, as shown in Fig. 1.

FIG. 1.—Sites of injection of mice of the first (left) and second (right) series.

The yield of sarcomas was as follows :

| | |
|---|---|
| 2-site series . . . . | 21 mice out of 50 developed sarcoma at single site ; |
| 4-site series . . . . | 29 mice out of 50 developed sarcoma, including 2 which had sarcoma at 3 sites, and 5 which had sarcoma at 2 sites. |

The figure of total tumours for the 2-site series is incomplete because some of the mice were killed early in the test for histological observations, otherwise the total of tumour-bearing mice would very probably have been much the same as in the 4-site series.

These results could suggest :

(1) that sarcoma formation at one site inhibits its development at another site ;
(2) the inhibiting factors can to some extent be overwhelmed if several sites are subjected to carcinogen simultaneously ;
(3) when the tumour begins to proliferate its rate of growth progressively increases and hence the mouse with a single tumour has to be killed before any of its other potential tumours have time to develop to detectable size ;
(4) 22 of the tumour-bearing mice in the 4-site series showed a single sarcoma which would support conclusion No. 3, and might lead to the inference that any one tissue is a mosaic showing different degrees of sensitivity to carcinogen.

Entirely different results were obtained by Nettleship and Henshaw (1943) and by Salaman (1958); Nettleship and Henshaw used urethane and described mouse lungs containing 60 or more foci of adenoma and Salaman painted " 101 " mice with benzopyrene and found the painted areas to contain numerous papillomas, in one case 80 tumours were counted on a single mouse.

EXPERIMENT 6

Berenblum (1954) has described experiments where considerable intervals, of the order of 10 months, were interposed between the initiating dose of carcinogen and the application of promotor (croton oil). In our experiments, described below, 3 series of 50 mice each (20 " 101 " strain, 20 C57, 10 BRO) were given two strokes interscapularly with a brushful of 0·5% DMBA solution, the treated area being of the order of 1–2 square centimetres. After 3 weeks, biweekly painting with 0·5% croton oil in acetone was begun in one series and continued as long as there were survivors. The second series was painted biweekly with the same croton oil solution beginning 3 months after the initiation, and the third series was so treated beginning 9 months after initiation. The yield of tumours was as follows :

| | | | |
|---|---|---|---|
| 3 week interval series | 36 papillomas of which | 27 became epitheliomas |
| 3 month ,, ,, | 32 ,, ,, ,, | 18 ,, ,, |
| 9 ,, ,, ,, | 25 ,, ,, ,, | 11 ,, ,, |

The survival rates were these :

| | Survivors | | | |
|---|---|---|---|---|
| | 6 months | 12 m. | 18 m. | 24 m. |
| 3 week interval series | 44 | 16 | 3 | 0 |
| 3 month ,, ,, | 44 | 26 | 11 | 0 |
| 9 ,, ,, ,, | 34 | 29 | 15 | 3 |

The latent periods of the papillomas (Table III) is estimated as the time from initiation to the formation of a fleshy nodule visible to the naked eye, say the size of a pin's head ; such an estimate is, of course, arbitrary, but is convenient for a comparative treatment of the results of the different experimental series.

The results suggest that :

(1) under the conditions of the experiment, and confirming Berenblum's (1954) work, initiation is in evidence for at least 9 months, which is of the order of $\frac{1}{2}$–$\frac{1}{3}$ of the life of the mouse ;

(2) since the time observed for a papilloma to develop into an epithelioma was very variable, and in many cases extended over 6 months, the lower total of epitheliomas in the 9 month series could be partly accounted for by the survival rate, the mice dying off before their papillomas became malignant. Furthermore, since there was comparatively little difference between the tumour yield in the 3 and 9 month series and since the epithelial cells must have been replaced many times in 9 months, it might be inferred that initiation takes place in permanent tissue (or perhaps in such tissue as the epidermal cells lining the hair follicles);

(3) the tumours which developed in the 3 and 9 month groups before promotion with croton oil were obviously induced by the single dose of DMBA ;

(4) promotion in all three groups is followed immediately by the appearance of tumours ; one can hardly suppose that the papillomas sprang instantaneously, almost, into being. It is more probable that the tissue which so rapidly becomes a visible papilloma is very well advanced in its progress to that condition ;

(5) the papillomas which appeared before the application of promotor are of particular interest showing that application of DMBA on one occasion only is sufficient to produce tumours.

(6) At first sight the results of experiment 6 are in accord with Berenblum theory with regard to the " average " latent period which when adjusted is practically the same for each series (3 week, 3 month and 9 month interval) ; these are (crude), 23, 39, and 66 weeks but when the interval is subtracted, these average latent periods become, 20, 26 and 25. Nevertheless, this conclusion is open to criticism : for example, it might be questioned whether " averages " for such widely dispersed ranges of figures do in fact have much value and secondly, taking into account the strain incidence, the figures for latent period could also suggest a modification of Berenblum's theory, namely, that initiation creates a pre-tumourous not a precancerous condition in the skin, i.e. the tumour cells have already been produced by the DMBA, and the few weeks which separate the commencement of the promotion treatment and the appearance of papillomas visible to the naked eye is a tumour-growth interval, *not* a promotion-process interval.

(7) The time of appearance of the tumours in Table III in relation to the time of initiation and promotion, and their density, i.e. close packing or otherwise, can be explained, or perhaps merely described by the following :

(a) the Berenblum-Rous theory is valid, i.e. the initial painting with potent carcinogen induces both fully developed tumours *and* areas of dormant cancer cells which more or less rapidly appear as tumours when promotor is applied.

(b) On promotion tumours appear over a period of almost 2 years, but the majority do so in loose groups according to which strain the mice belong ; the " 101 " strain tumours appear first, then the BRO strain and last the C57 strain tumours.

(c) It is remarkable that both in the 3-week interval series and in the 3-month series the majority of tumours appeared in the period 5–27 weeks after initiation (i.e. irrespective of whether promotion was begun at 3 weeks or at 3 months (13 weeks)) ; the preponderance of tumours in this period does not support Berenblum's " average latent period " which is of somewhat doubtful value for it is heavily weighted by the individual tumours of very long latent period ; moreover, this crowding of latent periods of both groups into the first 27 weeks strongly suggests that the essential carcinogenic process is taking place continuously and is only revealed, as it were, by the promotor.

(d) There is a suggestion that the last few tumours to develop in all three groups have been induced by croton oil *per se* and not by the DMBA-croton oil process.

(e) In all three series a concentration of tumours appears when promotion is instituted followed by a spasmodic development of tumours spaced a few weeks apart (from the 27th to the 96th week in the 3-week interval group, from the 33rd to the 97th week in the 3-month group and from the 58th to the 104th week in the 9-month group) ; this spasmodic development raises the question of why the tumours appear at such widely different latent periods even in the mice which are genetically homogeneous. The latent periods would be expected to show a normal curve of distribution, but such is far from the case. A correction for survivors at risk does not provide a solution, for the latent periods are as spasmodic when the survivors are more than 50% of the initial number as when the survivors diminish to $\frac{1}{10}$ or even fewer as is shown in Table IIIA. A solution might become available if the experiment were repeated on a much larger scale using say 500 mice instead of 50 mice per group. A hint is perhaps suggested by the sarcoma-induction

TABLE III.—*Experiment* 6

Latent period of appearance of papillomas (weeks) Each number represents one papilloma and its latent period

Initiation with DMBA
Promotion with croton oil

| 3-week interval series (i.e. 3 weeks between initiation and promotion | | 3-month interval series | | 9-month interval series | |
|---|---|---|---|---|---|
| Latent period | Strain | Latent period | Strain | Latent period | Strain |
| . . . (Promotion at 3 weeks) | | | | | |
| 5 | BRO | | | | |
| 6, 6, 6, 6, 6, 6, 6, 6, | 101, C57, C57, 101, 101, 101, 101, 101 | | | | |
| | | | | 7 | 101 |
| 8, 8, 8, 8, | } | | | | |
| 9, 9, 9, | } | 9, 9, | 101, BRO | 9, 9, | BRO, BRO |
| | } 101 | | | 10 | 101 |
| 11 | } | | | | |
| 12, 12, 12, | } | 12 | 101 | | |
| | } . . . | (Promotion) at 3 months) | | | |
| 14 | } | 14, 14 | } | | |
| | | | } | 16 | BRO |
| 17 | C57 | | } 101 | | |
| | | 18 | } | | |
| | | 19, 19, 19, | } | | |
| | | 20 | } | 20, 20 | 101, 101, |
| | | 21 | } | | |
| | | 22 | } BRO | | |
| 23 | C57 | | } | | |
| 26, 26, 26, | BRO, BRO, BRO | 26, 26, | } | | |
| 27 | BRO | 27, 27, 27 | } | | |
| 32 | C57 | 32 | BRO | | |
| | | 33 | 101 | | |
| 35 | BRO | 35 | C57 | | |
| | | 38 | BRO | | |
| 42 | C57 | | | . . . (Promotion at 9 months = 39 weeks) | |
| | | | | 44, 44 | 101, 101, |
| | | | | 46, 46 | 101, 101 |
| | | 47, 47 | C57, C57 | 47 | 101 |
| 48, 48 | C57, C57 | 48 | BRO | 48 | BRO |
| | | | | 49 | BRO |
| | | 52 | } | | |
| | | 56 | } | | |
| | | | } | 58 | 101 |
| | | 61 | } | 61 | BRO |
| 69 | C57 | 69 | } | 69 | BRO |
| | | 71 | } | | |
| 75 | BRO | | } C57 | | |
| | | 77 | } | 77, 77, 77, | } C57 |
| 78 | C57 | | } | 78 | } |
| | | | } | 80, 80 | BRO, BRO |
| | | 83 | } | | |
| 96 | BRO | | } | | |
| | | 97 | } | | |
| | | | | 104, 104 | BRO, BRO |

experiments described elsewhere in this paper, where threshold amounts of carcinogen were injected subcutaneously ; there the latent periods presented a normal distribution curve, but of course an added complication in such experiments lies in the fact that the sarcoma is detected when it is already palpable while the skin papilloma is detected at a very early stage when it is only about the size of a pin's head. To add to the complexities, the time required for growth of a tumour from pin-head to palpability and the time required from the stage of dormant-cell group to pin-head papilloma are still unknown factors.

TABLE IIIA.—*Experiment* 6

Table showing (1) latent periods, (2) number of tumour bearing mice at these times, (3) the corrections which might be applied for the decline in survivors at risk.

| 3-week interval series | | | 3-month interval series | | | 9-month interval series | | |
|---|---|---|---|---|---|---|---|---|
| Latent period (weeks) | No. of mice (tumours) | Correction | Latent period (weeks) | No. of mice (tumours) | Correction | Latent period (weeks) | No. of mice (tumours) | Correction |
| 5 | 1 | 0 | | | | | | |
| 6 | 8 | 0 | | | | | | |
| | | | | | | 7 | 1 | 0 |
| 8 | 4 | 0 | | | | | | |
| 9 | 3 | 0 | 9 | 2 | 0 | 9 | 2 | 50/49 |
| | | | | | | 10 | 1 | 50/48 |
| 11 | 1 | 50/49 | | | | | | |
| 12 | 3 | 50/49 | 12 | 1 | 50/48 | | | |
| 14 | 1 | 50/49 | 14 | 2 | 50/47 | | | |
| | | | | | | 16 | 1 | 50/47 |
| 17 | 1 | 50/48 | | | | | | |
| | | | 18 | 1 | 50/46 | | | |
| | | | 19 | 3 | 50/46 | | | |
| | | | 20 | 1 | 50/46 | 20 | 2 | 50/45 |
| | | | 21 | 1 | 50/46 | | | |
| | | | 22 | 1 | 50/46 | | | |
| 23 | 1 | 50/45 | | | | | | |
| 26 | 3 | 50/44 | 26 | 2 | 50/44 | | | |
| 27 | 1 | 50/44 | 27 | 3 | 50/44 | | | |
| 32 | 1 | 50/35 | 32 | 1 | 50/41 | | | |
| | | | 33 | 1 | 50/41 | | | |
| 35 | 1 | 50/34 | 35 | 1 | 50/40 | | | |
| | | | 38 | 1 | 50/38 | | | |
| 42 | 1 | 50/28 | | | | | | |
| | | | | | | 44 | 2 | 50/31 |
| | | | | | | 46 | 2 | 50/29 |
| | | | 47 | 2 | 50/26 | 47 | 1 | 50/28 |
| 48 | 2 | 50/17 | 48 | 1 | 50/26 | 48 | 1 | 50/28 |
| | | | | | | 49 | 1 | 50/28 |
| | | | 52 | 1 | 50/23 | | | |
| | | | 56 | 1 | 50/23 | | | |
| | | | | | | 58 | 1 | 50/19 |
| | | | 61 | 1 | 50/14 | 61 | 1 | 50/19 |
| 69 | 1 | 50/4 | | | | 69 | 1 | 50/15 |
| | | | 71 | 1 | 50/10 | | | |
| 75 | 1 | 50/3 | | | | | | |
| | | | 77 | 1 | 50/7 | 77 | 3 | 50/13 |
| 78 | 1 | 50/3 | | | | 78 | 1 | 50/12 |
| | | | | | | 80 | 2 | 50/9 |
| | | | 83 | 1 | 50/5 | | | |
| 96 | 1 | 50/1 | | | | | | |
| | | | 97 | 1 | 50/1 | | | |
| | | | | | | 104 | 2 | 50/2 |

(8) In the 9-month interval series, 7 tumours appeared within 10 weeks after the start of promotion, but 7 tumours also appeared in the first 20 weeks before promotion was begun, thus promotion in this series was of a quite different order of efficiency from that in the 3-week and 3-month interval series, which suggests that initiation largely declines somewhere between 3 and 9 months after its establishment.

EXPERIMENT 7

*Initiation and promotion effected by one application of DMBA*

The inferences suggested by experiment 6 are supported, to some extent, by the results of experiments about to be described : four series of 50 mice each (15 C57, 10 stock, 15 $F_2$ (Buffalo $\times$ C57), 10 BRO) were given a single painting with a " small " brushful of 0·5% DMBA, i.e. much less DMBA was used than in experiment 6. After 3 months, one series was then painted with 0·5% croton oil bi-weekly ; a second series was given the same croton oil treatment but not until 8 months after initiation, and the remaining two series remained untreated after the initiating painting with DMBA. Seventeen months after the start of the experiment, the yield of tumours was as follows :

| | |
|---|---|
| 3 month interval series (A) . . | 27 papillomas of which 8 became epitheliomas (24 survivors) |
| 8 month interval series (B) . . | 13 papillomas of which 3 became epitheliomas (35 survivors) |
| 1st no-croton series (C) . . | 7 papillomas of which 2 became epitheliomas (32 survivors) |
| 2nd no-croton series (D) . . | 7 papillomas of which 2 became epitheliomas (36 survivors) |

The latent periods are shown in Table IV.

These results suggest several conclusions, for example :

(1) As in experiment 6, one painting with 0·5% DMBA is sufficient to induce tumours, both papillomas and epitheliomas, i.e. a single application can initiate and promote ;

(2) again as in experiment 6, a number of tumours developed before promotor was applied but curiously not in series B ; moreover tumours also appeared in the series which were not treated with promotor at all (C and D), and these two series produced tumours much earlier than either of the two which received croton oil (A and B).

(3) The tumours developing late in series A and B appear to be due to the promoting action of the croton oil, or, which is more likely, they are due to croton oil alone, acting as a complete carcinogen. Since papillomas appeared in series C and D (no croton oil) in the period covered by the 40–54 weeks range, those which arose in this period in A and B might also have arisen without the intervention of croton oil, especially as there is a gap in B between the 54th week and the 71st when no papillomas were observed. If these suppositions are valid then it follows that after a single painting of a " small area " with DMBA the initiating stimulus is lost after somewhere between 3 and 8 months, possibly it is the initiated cells which are lost, not the stimulus.

### TABLE IV.—*Experiment 7*

Latent period of appearance of papillomas (weeks)
Each number represents one papilloma and its latent period

Initiation with DMBA ; Promotion with croton oil

| 3-month interval series (A) (i.e. 3 months between Initiation and Promotion) | | 8-month interval series (B) | | No-promotion series (C) | | No-promotion series (D) | |
|---|---|---|---|---|---|---|---|
| Latent period | Strain | Latent period | Strain | Latent period | Strain | Latent period | Strain |
| | | | | 3 | Stock | 3, 3, | Stock Stock |
| | | | | 4 | F2 | | |
| | | | | 6 | BRO | | |
| | | | | 7 | BRO | | |
| 12 | BRO | | | | | 12 | Stock |
| . . . (Promotion at 3 months = 13 weeks) | | | | | | | |
| 20, 20, | $F_2$, C57 | | | | | | |
| 23, 23 | Stock, Stock | | | | | | |
| 25 | C57 | | | | | | |
| 29 | Stock | | | | | | |
| 30 | Stock | | | | | | |
| 34 | $F_2$ | | | | | | |
| | | . . . (Promotion at 8 months = 36 weeks) | | 40 | Stock | 40 | $F_2$ |
| | | 42 | BRO | | | | |
| | | | | 45 | Stock | | |
| 46, 46 | $F_2$, $F_2$ | | | | | | |
| 48, 48 | $F_2$, BRO | 48 | Stock | | | | |
| 49, 49 | $F_2$, BRO | | | 49 | Stock | 49 | Stock |
| | | | | | | 50 | $F_2$ |
| 51, 51 | BRO, BRO | 51, 51 | $F_2$, BRO | | | | |
| 52 | Stock | 52 | BRO | | | | |
| | | 54, 54 | $F_2$, $F_2$ | | | 54 | Stock |
| 55 | Stock | | | | | | |
| 64 | BRO | | | | | | |
| 67 | BRO | | | | | | |
| | | 71 | $F_2$ | | | | |
| | | 72 | $F_2$ | | | | |
| 73 | BRO | | | | | | |
| 74 | $F_2$ | | | | | | |
| 78 | } | | | | | | |
| 80 | } C57 | 80 | C57 | | | | |
| 84 | } | 84 | $F_2$ | | | | |
| 88 | } | | | | | | |
| | | 94, 94 | BRO, BRO | | | | |

To a certain extent the latent period tables for experiments 6 and 7 agree, if the difference between the initiating doses of DMBA (one small stroke of a brushful of DMBA in experiment 7, and two strokes in experiment 6) be taken into account.

(4) The observations that referred to the range of latent periods of experiment 6 (section 6) apply also to the figures for latent periods in experiment 7, but with this difference, the crude average for series A and B (experiment 7) are 50 and 57, but if the initiation-promotion interval be subtracted, these averages become 37 and 19 a ratio of practically 2 : 1 which would provide support for the possibilities put forward in paragraph 3 namely that in series B of experiment 7 the papillomas were not in fact induced by the promoting action of croton oil or DMBA initiated cells, but were either largely due to croton oil alone or to DMBA acting alone. But again, any such possibility depends upon what value can be placed on latent period " averages ".

### EXPERIMENT 8

*Multi-stage experiment using benzopyrene*

Five series of 45 mice each (15 $F_2$ (C57 × Buffalo) strain, 10 BALB, 10 BRO, 10 Stock) were treated as follows : all five series were given a single painting with 0·5% benzopyrene (BP) instead of DMBA ; then, the first series (A) was painted biweekly with 0·5% croton oil, beginning 1 month after initiation, the second series (B) was painted biweekly with 0·5% croton oil, beginning 4 months after initiation, the third series (C) was painted biweekly with 0·5% croton oil, beginning 10 months after initiation; the fourth series (D) was not treated with croton oil, nor was the fifth series (E).

15 months after initiation the tumour yield was as follows :

| | | | | | |
|---|---|---|---|---|---|
| A series (1 month interval) | 8 | papillomas | giving | 1 | epithelioma |
| B series (4 ,, ,, ) | 5 | ,, | ,, | 1 | ,, |
| C series (10 ,, ,, ) | 4 | ,, | ,, | 1 | ,, |
| D series (no croton) | 0 | ,, | ,, | 0 | ,, |
| E series (no croton) | 1 | ,, | ,, | 0 | ,, |

The latent periods are shown in Table V. One feature of the results of this experiment is in agreement with experiment 7 and experiment 6 (DMBA), suggesting a gradual decline of initiated cell-groups (or of the initiated state) beginning 3 or 4 months after initiation ; moreover while the tumour yield refers to the respective potencies of the two carcinogens, the time for the beginning of the decline mentioned above could mean that the life span of the initiated cells is the determining factor in this phenomenon, any other explanation would probably involve even more tortuous concepts such as different degrees of initiation to which the transformed cells had been brought by the single application of carcinogen. The term " single application " implies that the tissue is being subject to the equivalent of a single dose of e.g. irradiation, but of course that is far from the case ; the carcinogen is certainly present in the skin for a few weeks and perhaps longer and must continue to act during that time. Except for a single papilloma in series C, there is a long interval between the start of promotion and the appearance of the

**Table V.—*Experiment* 8**

Latent period of appearance of papillomas (weeks)
Each number represents one papilloma and its latent period

Initiation with Benzopyrene

Promotion with croton oil

| 1-month interval series (A) (i.e. 1 month between Initiation and Promotion) | | 4-month interval series | | 10-month interval series (C) | | No promotion (series D) | | No-promotion (series E) | |
|---|---|---|---|---|---|---|---|---|---|
| Latent period | Strain | Latent period | Strain | Latent period | Strain | Latent period | Strain | Latent period | Strain |
| . . . (Promotion at 1 month = 4 weeks) | | | | | | | | | |
| | | . . . (Promotion at 4 months = 17 weeks) | | | | | | | |
| 20 | $F_2$ | | | | | | | | |
| 23 | $F_2$ | | | | | | | | |
| | | 42 | $F_2$ | | | | | | |
| 43 | Stock | | | | | | | | |
| 44 | Stock | 44 | Stock | | | | | | |
| 46 | Stock | | | | | | | | |
| | | | | . . . (Promotion at 10 months = 43 weeks) | | | | | |
| | | | | 53 | $F_2$ | | | | |
| 68, 68 | $F_2$, $F_2$ | 68 | $F_2$ | | | | | | |
| | | 72 | $F_2$ | | | | | | |
| 78 | $F_2$ | | | | | | | | |
| | | 84 | BALB | | | | | | |
| | | | | 85, 85, 85 | $F_2$, $F_2$, BALB | | | | |
| | | | | | | | | 91 | BALB |

earliest papilloma, a result which is quite different from the rapid development of tumours in experiment 6, and especially 7.

However, the results of this experiment (8) can also be interpreted in the light of experiment 10 below where croton oil is shown to be a complete carcinogen of low potency ; the tumours observed in series B and C and perhaps A of experiment 8 could have been induced in part, at least, by croton oil alone, the low yield being due to a combination of diminishing survivors and the long latent period required by croton oil ; if such is the case, then benzopyrene could not have carried out initiation under the conditions of the experiment.

It would not be easy to design an experimental test of the question which would satisfy all requirements ; the problem is complicated, for example, by the following factors : (a) potent carcinogens induce papillomas in mice after a single application ; (b) the promotor (croton oil) is itself a weak but complete carcinogen for the skin ; (c) the promotor is usually applied not once but repeatedly, sometimes of the order of a hundred times in many experiments on initiation-promotion.

To test what has been named the " reverse experiment ", mouse skin should therefore be first prepared with promotor applied say, 50 or 100 times before painting once with " initiator " (DMBA), i.e. the order of promotor-initiator should be reversed, but in addition the number of applications should be in proportion to their potency as complete carcinogens. Under such conditions and provided (which is dubious) that further complications have not been introduced, the additive character of promotion might be assessed. Possibly croton oil acts in the role, say, of a lubricant in a machine, i.e. the contribution made by the oil to the process is facilitating but is not additive to the energetics of the changes involved.

The latents periods of series A and B tumours (Table V) show remarkable similarity, apart from the two earliest papillomas in A ; this similarity is difficult to understand, it is as though the time when promotion was begun were unimportant and that the time of development of the tumours depended almost entirely upon the initiation stage.

### EXPERIMENT 9

*To find if hydrocarbons of low, but complete carcinogenic activity can show promoting effect for mouse skin initiated by DMBA*

Three series of 50 mice each were set up ; their composition was not as uniform as was desirable but consisted of the following strains :

| | | | | | |
|---|---|---|---|---|---|
| Series A : | 10 stock, | 10 " 101 ", | 10 BRO, | 10 BALB, | 10 C57 |
| ,, B : | ,, ,, | ,, ,, | ,, ,, | ,, ,, | ,, ,, |
| ,, C : | 15 ,, | | 25 BRO | | 10 C57 |

The mice were painted interscapularly as follows :

Series A : a single painting with 0·5% DMBA : after an interval of 3 weeks—biweekly painting with 0·25% 1,2 : 3,4,-dibenzanthracene ;
Series B : a single painting with 0·5% DMBA ; after an interval of 3 weeks—biweekly painting with solvent only ;
Series C : biweekly painting with 0·25% 1,2 : 3,4-dibenzanthracene (no previous treatment).

The tumour yields were as follows :

| | | | | | | |
|---|---|---|---|---|---|---|
| Series A : | 18 papillomas, | of which | 13 | became | epitheliomas |
| ,, B : | 11 ,, | ,, ,, | 7 | ,, | ,, |
| ,, C : | 3 ,, | ,, ,, | 0 | ,, | ,, |

These results could be interpreted thus :

(a) that the hydrocarbon of low carcinogenic potency (1,2 : 3,4-dibenzanthracene) appears to have some promoting effect upon initiated skin since the increased yield of papillomas and epitheliomas in series A over series B is more than the yield of tumours in series C which could imply that the promoting effect of this hydrocarbon is more obvious than the initiating capacity.

### EXPERIMENT 10

*Carcinogenic potency of croton oil*

The test of Initiation-Promotion theory depends upon the availability of pure initiators and pure promotors, or at least substances which are predominantly one or the other, but croton oil, the most important promotor, can be shown to have initiating capacity as well as promoting activity.

(a) A series of 80 mice (Stock, C57, BRO and " 101 " strains) were painted biweekly with 0·5% croton oil in acetone ; the tumour yield was as follows :

6 papillomas of which 2 became epitheliomas ; of the 6 papilloma 4 were at the painting site (the movements of the mice and their grooming spreads the painted material over the fur generally) ; of the 6 papillomas, 3 were in the 20 mice of " 101 " strain. The latent periods and the strains in which the tumours developed were as follows :

| Latent period (weeks) | Strain |
|---|---|
| 87 | " 101 " |
| 93 | " 101 " |
| 93 | " 101 " |
| 94 | BALB |
| 98 | C57 |
| 105 | BRO |

(b) croton oil was diluted with olive oil to give a 0·375% solution, at which concentration no appreciable ulceration was observed when the dilution was injected subcutaneously.

50 mice of C57 strain mice were given 0·2 c.c. of the dilution three times, at the start of the experiment, after 1 month and after 4 months. The yield was six tumours at the site of injection ; four were spindle celled sarcomas, and two were round celled tumours which were highly cellular, showed numerous mitoses and could therefore be classified as malignant. Croton oil can thus be considered a complete carcinogen.

SUMMARY

Experimental conditions, results and conclusions from studies on carcinogenesis in mice : (1) relation between amount of carcinogen and tumour response : (2) threshold doses of dimethylbenzanthracene and of benzopyrene ; (3) effect of altering vehicle for carcinogen, and of ageing in the mice ; (4) influence of volume of injected carcinogen solution ; (5) injection of carcinogen in a number of sites : (6) initiation and promotion ; tumour induction by croton oil.

This investigation has been supported by grants to the Chester Beatty Research Institute (Institute of Cancer Research : Royal Cancer Hospital) from the Medical Research Council and the British Empire Cancer Campaign for Research, and by the Public Health Service Grant No. CA-03188-08 from the National Cancer Institute, U.S. Public Health Service.

REFERENCES

BERENBLUM, I.—(1954) *Cancer Res.*, **14**, 471.
BOUTWELL, R. K.—(1964) *Prog. exp. Tumor Res.*, **4**, 207.
NETTLESHIP, A. AND HENSHAW, P. S.—(1943) *J. natn. Cancer Inst.*, **4**, 309.
ORR, J. W.—(1958) *Br. med. Bull.*, **14**, 99.
SALAMAN, M. J.—(1958) *Ibid.*, **14**, 116.